# Footwear for osteoarthritis of the lateral knee: protocol for the FOLK randomised controlled trial

**DOI:** 10.1186/s12891-020-03275-5

**Published:** 2020-04-15

**Authors:** Kade L. Paterson, Kim L. Bennell, Ben R. Metcalf, Penny K. Campbell, Jessica Kasza, Tim V. Wrigley, Rana S. Hinman

**Affiliations:** 1 Centre for Health, Exercise and Sports Medicine, Department of Physiotherapy, School of Health Sciences, Faculty of Medicine Dentistry & Health Sciences, The University of Melbourne, Melbourne, Australia; 2 School of Public Health and Preventive Medicine, Monash University, Melbourne, Australia

**Keywords:** Osteoarthritis, OA, Knee, Tibiofemoral, Footwear, Shoes, Clinical trial, RCT, Biomechanics, Pain

## Abstract

**Background:**

Structural features of lateral tibiofemoral (TF) joint osteoarthritis (OA) occur in up to half of all people with knee OA, and co-existing lateral TF OA is associated with worse knee pain in people with mixed compartmental knee OA. Clinical guidelines for management of knee OA advocate advice about appropriate footwear, yet there is no research evaluating which types of footwear are best for managing pain associated with lateral TF OA. Biomechanical evidence suggests that “motion-control” footwear, which possess midsoles that are stiffer medially compared to laterally, may shift load away from the lateral compartment of the knee and thus may reduce knee pain associated with lateral TF OA. The primary aim of this study is to compare the effects of motion-control shoes to neutral shoes on knee pain in people with predominantly lateral TF OA.

**Methods:**

This will be an assessor- and participant-blinded, two-arm, comparative effectiveness randomized controlled trial (RCT) conducted in Melbourne, Australia. We will recruit a minimum of 92 people with painful lateral TF OA from the community. Participants will be randomly allocated to receive either motion-control shoes or neutral shoes and will be instructed to wear their allocated shoes for a minimum of 6 h per day for 6 months. The primary outcome is change in self-reported knee pain on walking, measured using a numerical rating scale, assessed at baseline and 6 months. Secondary outcomes include other measures of knee pain, physical function, quality of life, participant-perceived change in pain and function, and physical activity levels.

**Discussion:**

This study will compare the efficacy of motion-control shoes to neutral shoes for people with painful lateral TF OA. Findings will be the first to provide evidence of the effects of footwear on knee pain in this important subgroup of people with knee OA and allow clinicians to provide accurate advice about the most appropriate footwear for managing pain associated with lateral TF OA.

**Trial registration:**

This trial has been prospectively registered by the Australian New Zealand Clinical Trials Registry on 15/11/2018 (reference: ACTRN12618001864213).

## Background

Osteoarthritis (OA) is a leading cause of pain and the 12th highest contributor to global disability [1]. The knee joint is often affected, and abnormal knee joint loading is central to OA pathogenesis [2, 3]. During walking, the ground reaction force vector typically passes medially to the knee joint centre, resulting in an external knee adduction moment. The knee adduction moment is a valid indicator of medial-to-lateral tibiofemoral (TF) joint load distribution [4], and it is this imbalance of force towards the medial compartment during load-bearing that likely explains why the medial TF compartment is more frequently affected by OA than the lateral compartment [5]. Nonetheless, structural features of lateral TF joint OA occur in 10–55% of cases of knee OA [5–9], and research has shown that co-existing lateral TF OA is associated with worse knee pain in people with mixed compartmental knee OA [10].

Non-surgical biomechanical treatment strategies are often advocated [11], and used clinically, to manage people with TF OA. Footwear is a promising avenue for self-management, given that foot position and motion influence medial-to-lateral TF load distribution. Accordingly, international clinical guidelines recommend clinicians provide advice regarding appropriate footwear to help manage the symptoms of knee OA [12, 13]. Unfortunately, all research evaluating footwear for OA has focussed on people with predominantly medial TF OA, and there are no RCTs evaluating the efficacy of footwear for people with predominantly lateral TF OA. This is a problem given that the biomechanics of people with lateral TF OA differ from those with medial TF OA [14], thus any evidence about the (in) effectiveness of biomechanical treatments for medial TF OA cannot be directly translated to the lateral TF OA. In support, leading international OA organisations have identified research on biomechanical interventions for specific OA subgroups as a key research recommendation [12].

In people with medial knee OA, a number of studies have shown that lateral wedge insoles [15], flat flexible shoes [16], and shoes with midsoles that are stiffer laterally than medially redistribute knee load *away* from the medial TF compartment towards the lateral compartment [17], albeit clinical effects are uncertain [18]. This is achieved via a lateral shift in the frontal plane ground reaction force-knee joint centre lever arm [19, 20] and an increase in lateral plantar pressures [21]. Likewise, footwear with midsoles that are stiffer medially compared to laterally, such as those with features that provide stability/support to the medial aspect of the foot, shift load *toward* the medial compartment [16, 22, 23], likely with a concomitant reduction in the lateral TF compartment. Such shoes are often referred to as “motion-control” or “stability” footwear. Although no study has directly investigated motion-control shoes, other research has shown that medially-wedged insoles, which also increase medial plantar pressures [24], reduce knee valgus malalignment in people with lateral knee OA [25]. In healthy populations, medially-wedged insoles [26] and medial arch supports [27] have also been shown to redistribute knee joint loading toward the medial TF compartment.

There is indirect RCT evidence supporting potential clinical improvements in people with lateral TF OA wearing motion-control footwear. A small RCT compared 30 women with lateral TF OA and bilateral knee valgus deformity wearing either medially wedged or neutral orthoses for 3–6 h/day for 8 weeks [28]. Compared to the neutral insoles group, the medially-wedged orthoses group showed greater improvements in pain with movement over 8 weeks, measured using a visual analogue scale (VAS; medially-wedged insoles: − 49% vs neutral insoles: − 6%, between group change *P* = 0.001), and in the Western Ontario McMasters University Osteoarthritis Index (WOMAC) total score (− 25% vs 3%, *P* = 0.001). Improvements in pain with movement and the total WOMAC score in the medially-wedged insole group exceeded minimal clinically important differences. However, the results of this small trial of medial wedge orthoses inserted into female participant’s own usual footwear cannot necessarily be extrapolated to shoes with in-built motion control features. There have been no RCTs testing the efficacy of any type of footwear on symptoms in people with lateral TF OA. As such, the efficacy of motion-control shoes for this condition remains unknown, and there is no evidence to inform clinical guidelines about which type of footwear is best for this important subgroup of patients with knee OA.

This study outlines the protocol for a RCT of footwear for people with symptomatic radiographic lateral TF OA. The primary aim of this RCT is to compare the effects of motion-control shoes and neutral shoes on knee pain in people with lateral TF OA. It is hypothesised that motion-control shoes will lead to significantly greater reductions in knee pain with walking compared to neutral shoes, when worn daily over 6 months, in people with lateral TF OA. Our secondary aim is to assess whether motion-control shoes improve other measures of pain, function, quality of life and physical activity compared to neutral shoes.

## Methods

### Study design

This study is a participant- and assessor-blinded, two-arm, comparative effectiveness RCT. The trial is being conducted at The University of Melbourne. It was prospectively registered with the Australian and New Zealand Clinical Trials Registry (ACTRN12618001864213) and is described using the Standard Protocol Items: Recommendations for Intervention Trials (SPIRIT) [29].

### Participants

Participants with painful predominantly lateral TF OA are being recruited from the community using print, radio, and social media advertisements, and via our existing network of clinicians and our volunteer database. The American College of Rheumatology clinical and radiographic criteria is being used to classify participants as having knee OA [30]. Participants are eligible for the study if they meet the following inclusion criteria:

i) aged ≥50 years;
ii) report knee pain on most days of the past month;
iii) report a minimum pain score of 4 on an 11-point numeric rating scale (NRS, with terminal descriptors of ‘no pain’ and ‘worst pain possible’) on average during walking over the previous week;
iv) have mild, moderate or severe (Grade 2–4) TF OA on x-ray according to the Kellgren & Lawrence (KL) grading system [31], one of the most widely used system for classifying radiographic severity of knee OA [32]; and
v) demonstrate a grade of lateral TF joint space narrowing greater than medial TF joint space narrowing, determined using a radiographic atlas [33] (where Grade 0 = no narrowing, 1 = mild narrowing, 2 = moderate narrowing, 3 = severe narrowing).

Participants will be excluded if they:

i) report knee pain for < 3 months;
ii) report recent knee surgery (in the past 6 months) or are planning to undergo surgery in the next 6 months;
iii) currently use foot orthoses, customised shoes, or ankle/knee braces;
iv) currently wear high heels, thongs or work boots for an extended period of time that would restrict their ability to wear the allocated study shoes for a minimum of 6 h per day;
v) have had a hip or knee replacement on their most painful side/knee;
vi) have had a high tibial osteotomy on their most painful knee;
vii) have had any injections in the knee joint in the past 3 months, or are planning an injection in next 6 months;
viii) report any other muscular, joint or neurological condition affecting lower limb function;
ix) report any systemic or inflammatory joint disease (eg rheumatoid arthritis);
x) currently use a gait aid, or plan to use one in the next 6 months;
xi) cannot understand written and/or spoken English;
xii) have a foot size outside the range of 6 to 12US for women and 7 to 13US for men; or
xiii) are unable to commit to the study requirements, such as wearing the study shoes, attending study appointments, or completing outcome measures.

### Procedure

All potential participants receive oral and written information about the purposes, potential risks and processes involved in the study from the Trial Coordinator. Informed consent is obtained from all participants by signing the consent form after the plain language statement has been read, and the salient information delivered verbally, and before proceeding with radiographic eligibility screening. Ethical approval has been obtained from the University of Melbourne Human Research Ethics Committee (HREC No. 1852787.1).

**Fig. 1:**
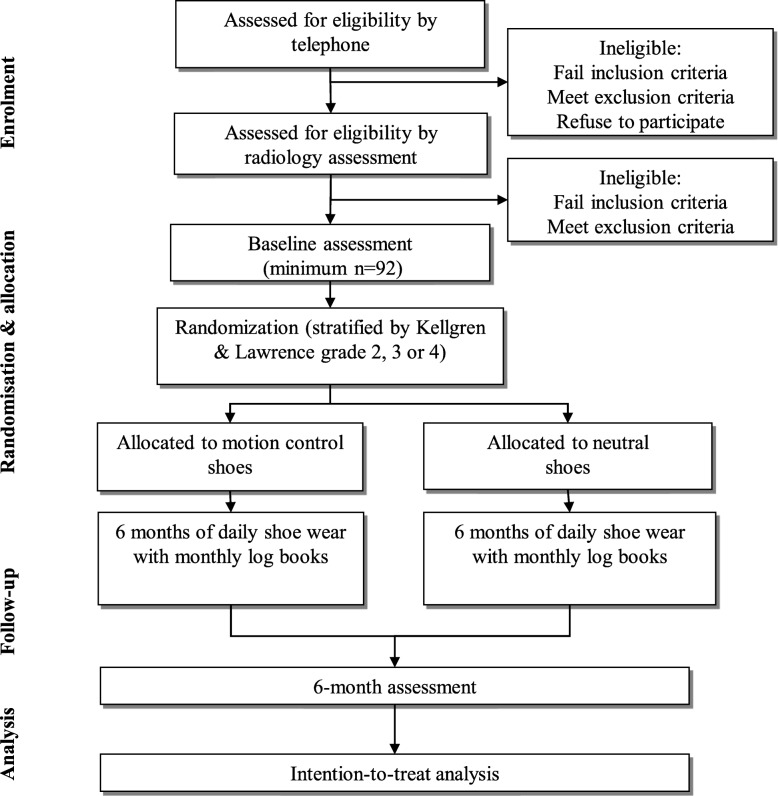
Flow diagram of study phases

The flow of participants through the study is outlined in Fig. 1. Potential volunteers are initially screened using an online form, and if they pass, are being contacted by the Trial Coordinator for further telephone screening. Those passing telephone screening are then booked for posteroanterior weightbearing x-rays at one of three radiology clinics in Melbourne, Australia for screening against radiographic eligibility criteria. If a participant has had a weightbearing posteroanterior or anteroposterior x-ray in the previous 2 years, and they are able to share the images with research staff for assessment, they are not required to undergo new x-rays. X-rays are graded by experienced research staff to confirm eligibility. In case a participant has bilaterally eligible knees, the most symptomatic knee is considered the study knee.

Participants are completing baseline assessments at the Department of Physiotherapy, The University of Melbourne. Six-month follow-up assessments (surveys) are being completed either on paper or electronically at home. Participants are also required to record adherence with wearing their study shoes for 1 week per month over the intervention period, using log books. If a participant fails to complete or return a log book or the follow-up survey, a researcher contacts them by phone or email as a prompt. Every effort is being made to minimize loss of data, including collection of the primary outcome over the telephone if necessary.

### Randomisation, blinding and allocation concealment

The randomisation schedule was generated by a biostatistician using permuted block sizes 6 to 12, and stratified by KL grades 2, 3 or 4. A researcher who is not involved with participant recruitment or assessment maintains the schedule on a password-protected website (REDCap) and reveals group allocation following baseline outcome assessment. This unblinded researcher is also responsible for measuring baseline participant characteristics in the laboratory, such as height, weight and foot posture, prior to fitting participants to their allocated shoes.

We use a process of limited disclosure to blind participants. We do not disclose any information to participants regarding the study hypotheses, or the shoe characteristics/model provided to participants in either group, to ensure blinding. Participants are told that we are comparing the effects of two types of readily available off-the-shelf walking shoes on knee OA symptoms in people with OA in the outer (lateral) compartment of the knee joint. Given participants are blinded to group allocation, and the primary and secondary outcomes are self-reported, this study is also considered assessor-blinded. Research staff administering and entering data, and the biostatistician performing statistical analyses, are also blinded.

### Footwear interventions

Table 1 describes the intervention (motion-control) and the comparator (neutral) shoes. We are comparing two different shoe models from the same manufacturer (ASICS) that differ in the amount of support/stability provided to the medial aspect of the foot, but appear similar visually.

**Table 1:**
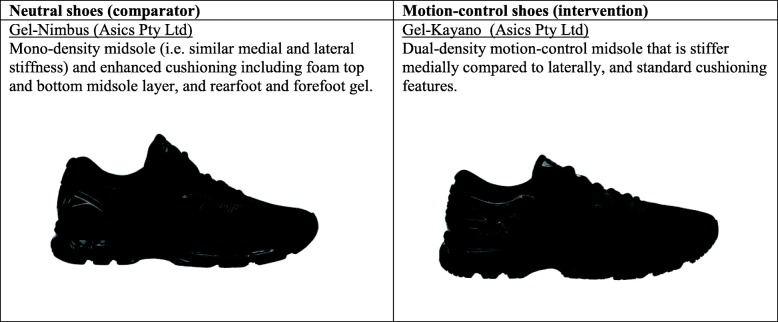
Features of the intervention and comparator shoes

We have previously consulted people with knee OA (*n* = 111; 68 women and 43 men) about their shoe preferences for a clinical trial. This is to ensure we only test shoes that are acceptable to consumers, and that potential participants are willing to wear them for at least 6 h per day over 6 months. Using a survey format with photographs of different shoe options and colours, we presented a range of motion-control shoes to the consumer panel for consideration. Findings showed that the Gel-Kayano (ASICS) shoes were a popular choice, and that black was overwhelmingly the preferred colour. Thus, we chose these as our intervention shoes. The predominant motion-control feature of the Gel-Kayano shoes is a dual-density ‘motion-control’ midsole that is stiffer medially compared to laterally. The comparator neutral shoe (Gel-Nimbus) was selected by the research team from other ASICS shoe models on the basis of being i) a neutral shoe with similar weight and structural characteristics to the intervention shoe, except for the dual-density motion-control feature and; ii) similar in appearance to the intervention shoe to ensure participant acceptability and willingness to wear.

Participants are fitted with, and provided, a pair of their allocated shoes to take home. Participants are instructed to initially wear the shoes for 2 h on the first day only, and then to increase wear time by 2 h/day. We advise participants that they should be wearing their allocated shoes as much as possible every day by the end of the first week, aiming for a minimum of 6 h/day for 6 months. No study has investigated the minimum amount of time required to wear shoes to achieve a clinical effect for people with knee OA, therefore we chose 6 h as this was shown to be feasible in our previous knee OA footwear RCT [34], and likely provides enough time for a therapeutic effect.

### Outcome measures

Table 2 describes the schedule of enrolment, interventions and the outcome measures for this study according to SPIRIT recommendations [29].

**Table 2:**
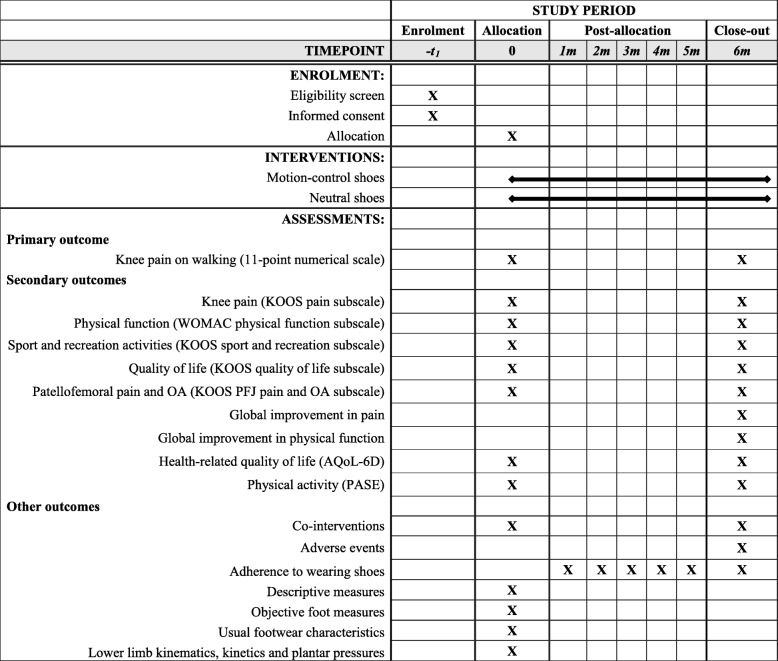
Schedule of enrolment, interventions and assessments

*WOMAC* Western Ontario and McMaster Universities Osteoarthritis Index, *KOOS* Knee injury and osteoarthritis outcome score, *PFJ* Patellofemoral joint, *OA* Osteoarthritis, *PASE* Physical activity scale for the elderly, *AQoL-6D* Assessment of Quality of Life instrument (version 6D)

#### Primary outcome

##### Knee pain on walking

Change in average pain on walking in the last week using an 11-point NRS with terminal descriptors ‘no pain’ (score = 0) and ‘worst pain possible’ (score = 10) from baseline to 6 months. This is a valid and reliable OA outcome measure [35], and is recommended for knee OA clinical trials [36].

#### Secondary outcomes

Secondary outcomes are assessed at baseline and at 6 months unless indicated otherwise. These include:

#### Physical function subscale of the WOMAC

The Western Ontario and McMaster Universities (WOMAC) Osteoarthritis Index (Likert version 3.1) is used to assess limitations with physical functioning [37]. The self-reported tool is a disease-specific instrument which has established validity, reliability and responsiveness in an extensive range of OA studies [38]. The subscale contains 17 questions on knee function over the past week, with Likert response options from ‘no dysfunction’ (score = 0) to ‘extreme dysfunction’ (score = 4). Total score ranges from 0 to 68, with higher scores indicating worse function. WOMAC scores will be extracted from the Knee Injury and Osteoarthritis Outcome Score (KOOS) questionnaire [39].

#### Pain subscale of the KOOS

The pain subscale of the KOOS is scored using nine questions regarding knee pain over the previous week, with Likert response options for each question ranging from none (score = 0) to extreme (score = 4) [39]. Scores are then transformed to provide an overall value that ranges from 0 to 100, with 0 representing extreme knee pain and 100 representing no knee pain.

#### Sport and recreation subscale of the KOOS

The sport and recreation subscale is assessed using five questions on function during sport and recreational activities over the previous week [39]. Likert responses for each question range from none (score = 0) to extreme (score = 4) [39]. Scores are then transformed to provide an overall value that ranges from 0 to 100, with 0 representing extreme problems with sport and recreation and 100 representing no problems with sports and recreation.

#### Quality of life subscale of the KOOS

This subscale is assessed using four questions on knee-related quality of life experienced in the previous week [39]. There are five Likert response options for each question, ranging from none (score = 0) to extreme (score = 4) [39]. Scores are then transformed to provide an overall value that ranges from 0 to 100, with 0 representing extreme problems with quality of life and 100 representing no problems with quality of life.

#### Patellofemoral pain and OA subscale of the KOOS

The patellofemoral pain and OA subscale includes 11 questions on knee pain and function experienced in the last week, each with five Likert response options, ranging from none (score = 0) to extreme (score = 4) [39]. Scores are then transformed to provide an overall value that ranges from 0 to 100, with 0 representing extreme patellofemoral problems and 100 representing no patellofemoral problems.

#### Participant-perceived global change

Participants rate their overall global change in pain and change in physical function over the 6 months since commencing the study, using a 7-point Likert scale with terminal descriptors of ‘much worse’ to ‘much better’ [40]. Participants reporting that they are ‘moderately better’ or ‘much better’ are classified as improved.

#### Health-related quality of life

Health-related quality of life is evaluated using the Assessment of Quality of Life (AQoL) (version AQoL-6D) [41]. The AQoL-6D contains 20 items that assess independent living, mental health, relationships, pain, coping and senses. Total scores range from − 0.04 to 1.00, with higher scores indicating better quality of life.

#### Physical activity levels

Physical activity over the previous week is assessed using the Physical Activity Scale for the Elderly (PASE) [42]. Total PASE scores range from 0 to over 400, with higher scores indicating greater physical activity.

#### Other measures

##### Co-interventions

Participants self-report co-intervention use (medications for knee pain and any other treatments for knee OA) at baseline and 6-months.

##### Adverse events

Adverse events (any problem experienced in the study knee or elsewhere in the body as a result of wearing the study shoes) are self-reported by participants at 6 months, and the proportion experiencing adverse events described along with the nature of the adverse events.

##### Treatment adherence

For each of 7 consecutive days each month of the 6-month intervention, participants record the number of hours that they wore their allocated shoes in log books. At the 6-month follow-up assessment, participants also rate their perceived overall level of adherence with wearing their allocated shoes (for a minimum of 6 h per day over the previous 6 months) on an 11-point NRS (with terminal descriptors of ‘shoes not worn all’ and ‘shoes worn completely as instructed’). Finally, participants also indicate whether they stopped wearing their allocated shoes during the 6 months on a categorical scale (Yes or No) at the 6-month follow-up. Participants who respond ‘Yes’ are required to describe when and why they stopped wearing their study shoes, and this will be reported descriptively.

##### Descriptive measures

These are recorded at baseline, and include height, body mass and body mass index; age; gender; duration of symptoms; radiographic disease severity (measured using the KL scale [31]); anatomical knee alignment (measured from the knee x-ray) [43]; current employment status; expectation of treatment outcome (rated on a 5-point ordinal scale with anchors of “no effect at all” to “complete recovery”); and self-efficacy (via the Arthritis Self Efficacy Scale [44]).

##### Objective foot measures

Objective foot measures are assessed at baseline, including the Foot Posture Index (FPI [45]), Foot Mobility Magnitude [46], and navicular drop [47]. In-shoe regional foot pressure patterns (Novel Pedar, Munich, Germany) are assessed following randomisation whilst walking in usual footwear and allocated study shoes, in random order.

##### Usual footwear characteristics

Motion-control characteristics of each participant’s most commonly worn shoes are recorded at baseline using the relevant items of the Footwear Assessment Tool [16]. Number of participants and proportion of participants’ usual shoes with motion-control features will be reported.

##### Lower limb kinematics and kinetics

Participants are assessed following randomisation walking barefoot and in their allocated shoes, presented in random order, while 3-dimensional gait analysis is performed. Gait analysis will only be completed on participants with a BMI < 36 kg/m^2^ due to difficulties performing gait analysis in people with a high BMI. A variety of biomechanical measures will be extracted, including parameters of the knee adduction/abduction moment (impulse and peaks). Changes in biomechanical parameters from the barefoot test condition to the allocated shoe condition will be compared between the two trial arms [16, 48]).

### Sample size calculations

We aim to detect the minimal clinically important difference on the primary outcome between groups (1.8 (out of 10) for NRS pain [49]). We conservatively assume a between-participant standard deviation of 2.7 units and a baseline to 6-month correlation of 0.21 based on our previous trials [34, 50]. Using analysis of covariance adjusted for baseline score, we need a minimum of 46 per arm to achieve 90% power to detect the minimal clinically important difference in pain. Allowing for 15% loss to follow up, we aim to recruit 55 participants per group. However, due to slow recruitment of participants with lateral TF OA (given the lower prevalence of lateral compared to medial TF OA), we will recalculate the sample size based on an updated attrition rate after 92 participants have been enrolled in to the study. The proportion of participants who have provided the primary outcome of NRS pain during walking at 6 months when the 92nd participant is enrolled will be calculated (pooled across the study arms). The sample size will then be revised based on this new attrition rate: if *p* patients are found to not have provided 6-month outcomes, the sample size required for each arm will be updated to 46/(1-*p*).

### Statistical analyses

A biostatistician will analyse blinded data. Main between-group comparative analyses will be performed using intention-to-treat, and multiple imputation will be used if more than 5% of the primary outcome is missing. Between-group differences in the mean change in the primary outcome of pain (baseline minus follow-up) will be compared using linear regression models, adjusted for baseline values of the primary outcome and the stratifying variable of KL grade. Continuous secondary outcomes will be analysed using similar methods. Risk differences, calculated from fitted logistic regression models, will be used to compare improvements in global change across groups. A sensitivity analysis will estimate treatment effects assuming full adherence to wear of the shoes (average of 6 h/day for 6 months, based on log-book data), using an instrumental variables approach [51]. Standard diagnostic plots will be used to check model assumptions.

To assess whether the effect of shoe allocation on the primary outcome is moderated by any of KL grade, FPI score, knee alignment, or baseline KOOS patellofemoral pain and OA score, appropriate interaction terms between randomised group and each of these variables will be included in regression models for the primary outcome, for each potential effect modifier separately.

### Timelines

Ethics approval was obtained from the University of Melbourne Human Research Ethics Committee in November 2018. Recruitment commenced in November 2018 and will be completed in February 2021. The trial is expected to be completed by August 2021 when all participants are due to have completed 6-month follow-up.

## Discussion

This will be the first RCT to test the efficacy of footwear for managing pain associated with predominantly lateral TF OA. We hypothesise that motion-control shoes will reduce knee pain more than neutral shoes over 6 months. Footwear is a promising self-management biomechanical treatment for people with knee OA, however all previous research has evaluated footwear in samples of people with predominantly medial TF OA. Findings from these studies cannot be applied to people with lateral TF OA, given that previous research has shown that people with lateral TF OA walk with different biomechanics to those with medial TF OA. There is plausible biomechanical evidence that motion-control shoes may shift load away from the lateral TF compartment, and thus may potentially improve pain, in people with lateral knee OA. Findings from the FOLK clinical trial will provide the first ever evidence about the effects of any type of footwear for people with lateral TF OA, and will thus help inform clinical guidelines about which types of footwear are optimal for managing symptoms in this important subgroup of people with OA.

## Data Availability

The datasets used and/or analysed during the current study will be made available from the corresponding author on reasonable request.

## Abbreviations

AQoL: Assessment of quality of life
CONSORT: Consolidated standards of reporting trials
FPI: Foot posture index
HREC: Human Research Ethics Committee
KL: Kellgren & Lawrence
KOOS: Knee injury and osteoarthritis outcome score
NRS: Numerical rating scale
OA: Osteoarthritis
PASE: Physical activity scale for the elderly
RCT: Randomised controlled trial
SPIRIT: Standard Protocol Items: Recommendations for Intervention Trials
TF: Tibiofemoral
WOMAC: Western Ontario and McMaster Universities
